# Transcriptomic and lipidomic analysis of an EPA-containing *Nannochloropsis* sp. PJ12 in response to nitrogen deprivation

**DOI:** 10.1038/s41598-019-41169-2

**Published:** 2019-03-14

**Authors:** Jibei Liang, Fang Wen, Jianhua Liu

**Affiliations:** 1 Ocean College, Zhejiang University, Zhoushan, ZJ316000 China; 2 Ocean Research Center of Zhoushan, Zhoushan, ZJ316021 China

## Abstract

To understand genes involved in neutral lipid accumulation upon nitrogen deprivation (ND) in a novel isolate of *Nannochloropsis* sp. PJ12, we performed comparative transcriptomic and lipidomic analyses of cells under ND and NR (nitrogen replete) conditions. Transcriptomic profiling indicated that, while enzymes involved in TCA cycle in PJ12 under ND condition were upregulated compared to that under NR condition, those involved in Calvin cycle and glycolysis under ND condition were downregulated. Furthermore, we showed that enzymes involved in fatty acid synthesis and glycerolipid synthesis were downregulated but not β-oxidation. Lipidomic profiling indicated that, while the level of neutral lipids in ND cells was increased compared to that of NR cells, level of photosynthetic membrane-lipids DGDG and PG was decreased. Taken together, our analysis indicated that TAG accumulation is attributed to the modification of membrane lipids derived primarily from “prokaryotic” pathway and secondarily from “eukaryotic” pathway based on the 16:X or 18:X fatty acid at the *sn*2 position of the glycerol backbone. We propose that two-phase (NR-ND) growth is ideal for biomass and biofuel production because ND reduces cell growth rate due to the loss of photosynthetic membrane and decreased quantum yield.

## Introduction

Microalgae are unicellular photosynthetic organisms capable of converting carbon dioxide into sugar. Oleaginous microalgae appear to store their carbon source in a form of neutral lipids or triacylglycerol (or TAG) such as *Nannochloropsis* spp. that are found in marine environment and also in fresh and brackish water^1^. *Nannochloropsis* spp. are considered promising algae for production of biodiesel^2,3^ and value-added molecules such as eicosapentaenoic acid (EPA)^4,5^. However, current algal strains are thought to be unsuitable for economic production of biofuels^6^ and require genetic improvement^7^.

A number of draft genome sequences of the *Nannochloropsis* spp. were published^7–11^. Molecular methods for manipulating genome sequences such as DNA transformation^8,12^, targeted gene knockout through homologous recombination^12^ and Crispr-Cas9-mediated genome editing^13,14^ have been established. Studies show that nitrogen deprivation (ND) is one of the most effective methods to increase neutral lipid storage in algae including *Nannochloropsis* spp.^3,15–22^.

Several transcriptomic analyses in *Nannochloropsis* spp. are performed for understanding genes responsible for induction of neutral lipids accumulation under ND condition. While the content of lipids increased, Carpinelli *et al*.^11^ detect no differential transcription of genes involved in lipid biosynthesis in *N*. *gaditana* B-31 upon ND, besides others. However, Li *et al*.^23^ show the upregulation of genes involved in Kennedy pathway including some isoforms of diacylglycerol acyltransferase (DGAT), though other isoforms of DGAT are downregulated^23^. Based on the prediction of subcellular localization of various enzyme isoforms involved in lipid biosynthesis together with lipidomic analysis, they propose that TAG accumulation in *N*. *oceanica* IMET1 under ND condition is a result of upregulation of lipid biosynthesis at multiple subcellular compartments^23^. However, silico prediction of subcellular localization is not always unambiguous^24–28^.

In photosynthetic organisms, free fatty acids such as palmitic acid (C16:0), stearic acid (C18:0), and oleic acid (C18:1n-9) are synthesized by type II fatty acid synthase (FAS II) in the plastid^29^. While some of the synthesized free fatty acids (FFA) are first assembled into diacylglycerol (DAG) and then into photosynthetic membrane lipids such as digalactosyldiacylglycerol (DGDG) and monogalactosyldiacylglycerol (MGDG) on the plastid membrane, some FFA are transported to endoplasmic reticulum (ER) where they are assembled into DAG and subsequently glycerophospholipids like phosphatidylcholine (PC) and phosphatidylethanolamine (PE)^29^. DAG in both compartments can convert into TAG using acyl-CoA-dependent and/or -independent approach^29^.

Subcellular localization of various enzyme isoforms can be deduced through sequence homologous comparison with those whose subcellular localization is known or using algorithm to predict subcellular compartments^30,31^. Wang *et al*.^32^ have found elevated number of enzyme isoforms involved in lipid biosynthesis compared to other algae and proposed that the high number of isoforms are linked to the high-level TAG accumulation in *Nannochloropsis* spp. Related to this issue, Nobusawa *et al*.^33^ have examined the subcellular localization of the four extraplastidic lysophosphatidic acid acyltransferases (LPAT or LPAAT) in *N*. *oceanica* NIES-2145 under ND condition and found that two out of four LPATs are associated with perimeter of lipid droplet under ND condition, indicating that functional involvement of various isoforms is complex.

It is known that in algae and plant, glycerolipids are synthesized by both “eukaryotic” and “prokaryotic” pathways^23,29,34–36^. Glycerolipids synthesized by the former and latter pathways are characterized with fatty acids 18:X and 16:X at *sn2* position of the glycerol backbone, respectively^23,29,31,34–36^. Lipidomics using liquid chromatography coupled with tandem mass spectrometry (LC-MS/MS) technique are able to determine levels of various classes of lipids and their associated molecular species of fatty acids including those at the *sn2* position on a large scale. Using both transcriptomic and lipidomic methodologies, pathways responsible for TAG accumulation in IMET1 under ND condition are readily revealed^23^.

In this study, we applied transcriptomic and lipidomic methodologies to investigate pathways responsible for TAG biosynthesis under ND condition in a novel isolate of *Nannochloropsis* sp. PJ12. We find that majority of TAG accumulates in PJ12 upon ND is attributed to the modification of membrane-lipids derived from both “prokaryotic” and “eukaryotic” pathway and associated with the decrease of quantum yield. Our analysis implies that production of TAG in algae would be benefited from using the two-phase growth scheme in which cultures are allowed reaching to the maximum density quickly under NR condition and then shift to ND condition for TAG accumulation through modification of membrane-lipids including photosynthetic membrane-lipids.

## Results

### Nitrogen deprivation induces the level of total lipids in a novel isolate *Nannochloropsis* sp. PJ12

**Figure 1:**
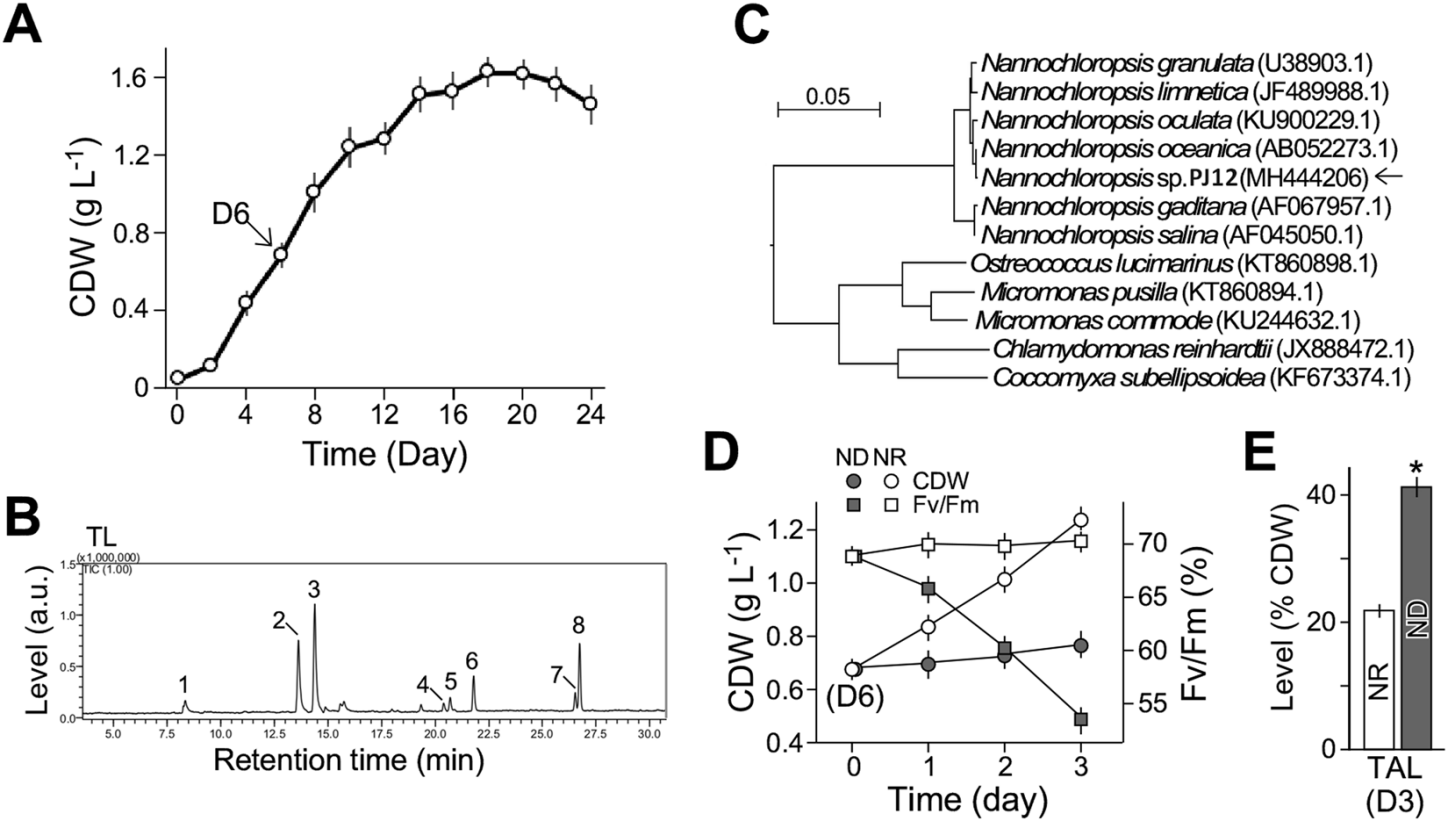
Characterization of a novel isolate *Nannochloropsis* sp. PJ12. (**A**) Growth curve analysis. X- and Y-axes indicate time (day) and cell dry weight (CDW, g L^−1^), respectively. Cell samples at day 6 as indicated were used for medium shift analysis. Average and standard deviation based on three repeats are shown. (**B**) Analysis of total lipids as fatty acid methyl esters (FAME) in cells under NR condition. Numbered fatty acids are described in text. (**C**) DNA-sequence-based phylogenetic tree analysis of *Nannochloropsis* sp. PJ12. Arrow indicates the position of PJ12. (**D**) Cell growth and photosynthetic yield (Fv/Fm) after medium shift to fresh nitrogen-depleted (ND) and nitrogen-replete (NR) media (*n* = 3). (**E**) Level of total lipids based on gravimetric analysis. Asterisk indicates the significant differences between levels in ND and NR cells (*n* = 3).

A microalgal isolate PJ12 from the area of Penjin, Liaoning province, China was obtained after a serial dilution from an outdoor culture pond (see Materials and Methods). PJ12 grew well in f/2 medium^37^ under continuous illumination of 50 µM photon m^−2^ s^−1^(see Materials and Methods) (Fig. 1A). Total acyl lipid contents under nitrogen-replete (NR) condition reached an average (*n* = 3) of 22% of cell dry weight (CDW) at mid-log phase based on gravimetric analysis.

GC-MS analysis of the total acyl lipid as fatty acid methyl esters (FAME) showed that PJ12 isolate contained eight major types of acyl-lipids: (1) myristic acid C14:0, (2) palmitoleic acid C16:1n-5, (3) palmitic acid C16:0, (4) linoleic acid C18:2n-6, (5) oleic acid C18:1n-9, (6) stearic acid C18:0, (7) arachidonic acid C20:4n-6, and (8) eicosapentaenoic acid (EPA) C20:5n-3 (Fig. 1B). Phylogenetic analysis using ClustalX and NJ tree^38^ based on the 18 S rDNA sequence derived from PJ12 (see Materials and Methods) suggested that PJ12 isolate belonged to a member of *Nannochloropsis* sp. with the highest similarity to that of *N*. *oceanica* (99.8% identity) (Fig. 1C).

To investigate the alteration of lipid contents between *Nannochloropsis* sp. PJ12 cells under NR condition and nitrogen-depleted (ND) condition, mid-log-phase cells at day 6 were subjected to growth medium shift: one half of the sample was cultivated in f/2 medium without nitrogen supplement (ND) and the other half was in f/2 with nitrogen (NR) as control (*n* = 3) (see Materials and Methods) (Fig. 1D). Cell growth and quantum yield were monitored after medium shift using gravimetric method and pulse amplitude modulation (PAM) technology, respectively (see Materials and Methods). We found that CDW was slightly increased, meanwhile quantum yield dramatically decreased based on an estimation using maximal PSII quantum yield (Fv/Fm) upon shift to ND medium (Fig. 1D, see filled signs). In contrast, CDW continued to rapidly increase and quantum yield remained unchanged after shift to NR medium (Fig. 1D, see open signs). Total acyl lipids as fatty acid methyl esters (FAMEs) was found to be ~41% and 22% of CDW under ND and NR conditions, respectively (Fig. 1E). These results indicated that ND induced the lipid accumulation in the EPA-containing *Nannochloropsis* sp. PJ12 strain.

### Cell size increases but mass density decreases upon nitrogen deprivation in *Nannochloropsis* sp. PJ12

**Figure 2:**
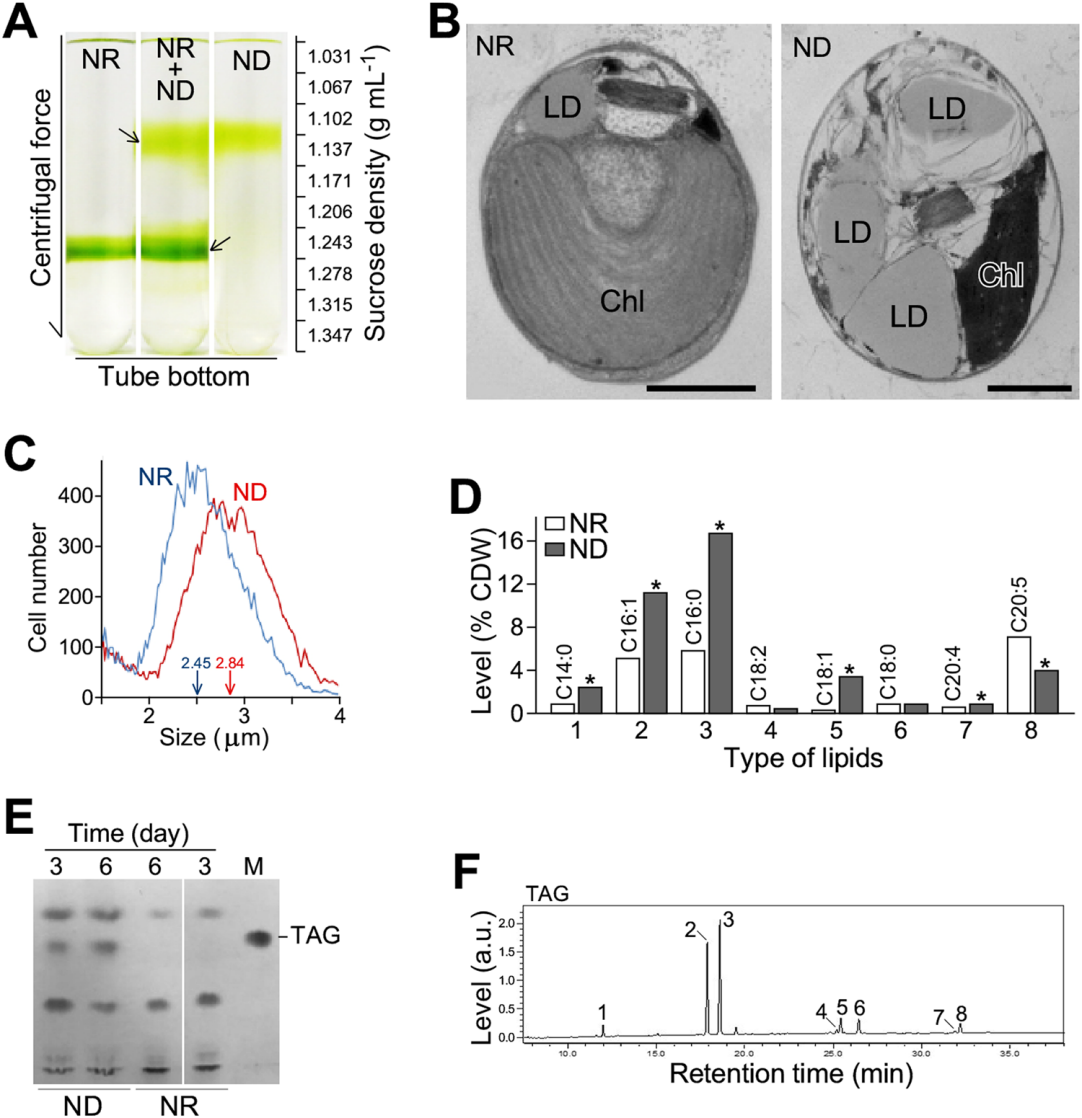
Cell mass density, size, and acyl-lipid level in PJ12 cells under ND and NR conditions. (**A**) Cell mass density based on step-wise sucrose gradient analysis. (**B**) Transmission electron microscopic analysis of ND and NR cells. LD and Chl indicate lipid droplet and chloroplast, respectively. One micrometer scale is indicated. (**C**) Cell sizes under ND and NR conditions. (**D**) Levels of acyl-lipids as FAMEs from a unit of cell dry mass under ND and NR conditions (*n* = 3). (**E**) Thin-layer chromatographic (TLC) analysis of total lipids under ND and NR condition. Position of triacylglycerol (TAG) is indicated. (**F**) Analysis of acyl-lipids from TAG as FAME in cells under ND condition. Numbered fatty acids are identical to Fig. 1B.

We subsequently applied a step-wise sucrose gradient centrifugation method to investigate cell mass density three days after medium shift to ND and NR (see Materials and Methods). The analysis indicated that the cell mass density under NR condition was between 1.243 g mL^−1^ and 1.278 g mL^−1^ (Fig. 2A). It was approximated to 1.261 g mL^−1^, the average of the two neighboring sucrose solution densities. On the other hand, cell mass density under ND condition was reduced by 12% between 1.102 g mL^−1^ and 1.137 g mL^−1^ (based on average of 1.120 g mL^−1^) compared to that of NR. This could be a result of lipid droplet accumulation in ND cells. To test this possibility, cells were subjected to transmission electron microscopic (TEM) analysis. The result showed that the size and number of lipid droplets in ND cells were greater than that in NR cells (Fig. 2B), supporting the hypothesis.

We noted that the size (or diameter) of cells under ND condition was also slightly greater than that under NR condition (median diameter: 2.84 μm versus 2.45 μm) (Fig. 2C). After converting to volume, the median cell volume under ND condition was found to be 50% greater more than the cell volume under NR condition (median volume: 12 μm^−3^ versus 7.7 μm^−3^). This result would suggest that, the identical amount of cell dry weight in ND and NR conditions could be produced by different numbers of cells. Hence, lipid levels were estimated based on per cell (or million cells) instead of per CDW.

Subsequently, we analyzed the alteration of various types of fatty acid levels under ND and NR conditions. GC analysis of the total acyl lipid as FAMEs showed that levels of myristic acid (C14:0), palmitoleic acid (C16:1n-5), palmitic acid (C16:0), oleic acid (C18:1n-9), and arachidonic acid (C20:4n-6) under ND condition was significantly increased compared to that of NR condition (fold change > 1.5-fold, p-value < 0.05), while content of EPA (C20:5n-3) was clearly decreased (fold change > 1.5-fold, p-value < 0.05) (Fig. 2D, Supplemental Fig. S2). Levels of linoleic acid (C18:2n-6) and stearic acid (C18:0) were hardly altered. Thin-layer chromatographic (TLC) analysis indicated that level of triacylglycerol (TAG) three days after medium shift to ND was increased by 5-fold higher than that under NR condition (Fig. 2E). We noted that TAG level was further increased by 20-fold six days after medium shift. This result suggested that TAG synthesis had not been stopped in cells three days after shifting to ND medium, suitable for transcriptomic and lipidomic profiling analysis. GC analysis of acyl lipids as FAMEs showed that major fatty acids in TAG were palmitoleic acid and palmitic acid (Fig. 2F). We also noted that levels of EPA in TAG under ND condition were lower than that of total acyl lipids under NR condition (see Fig. 2D), indicating that EPA was not a preferable component in neutral lipid.

### Photosynthetic membrane lipids contribute to the accumulation of neutral lipids upon nitrogen deprivation in PJ12 cells

**Figure 3:**
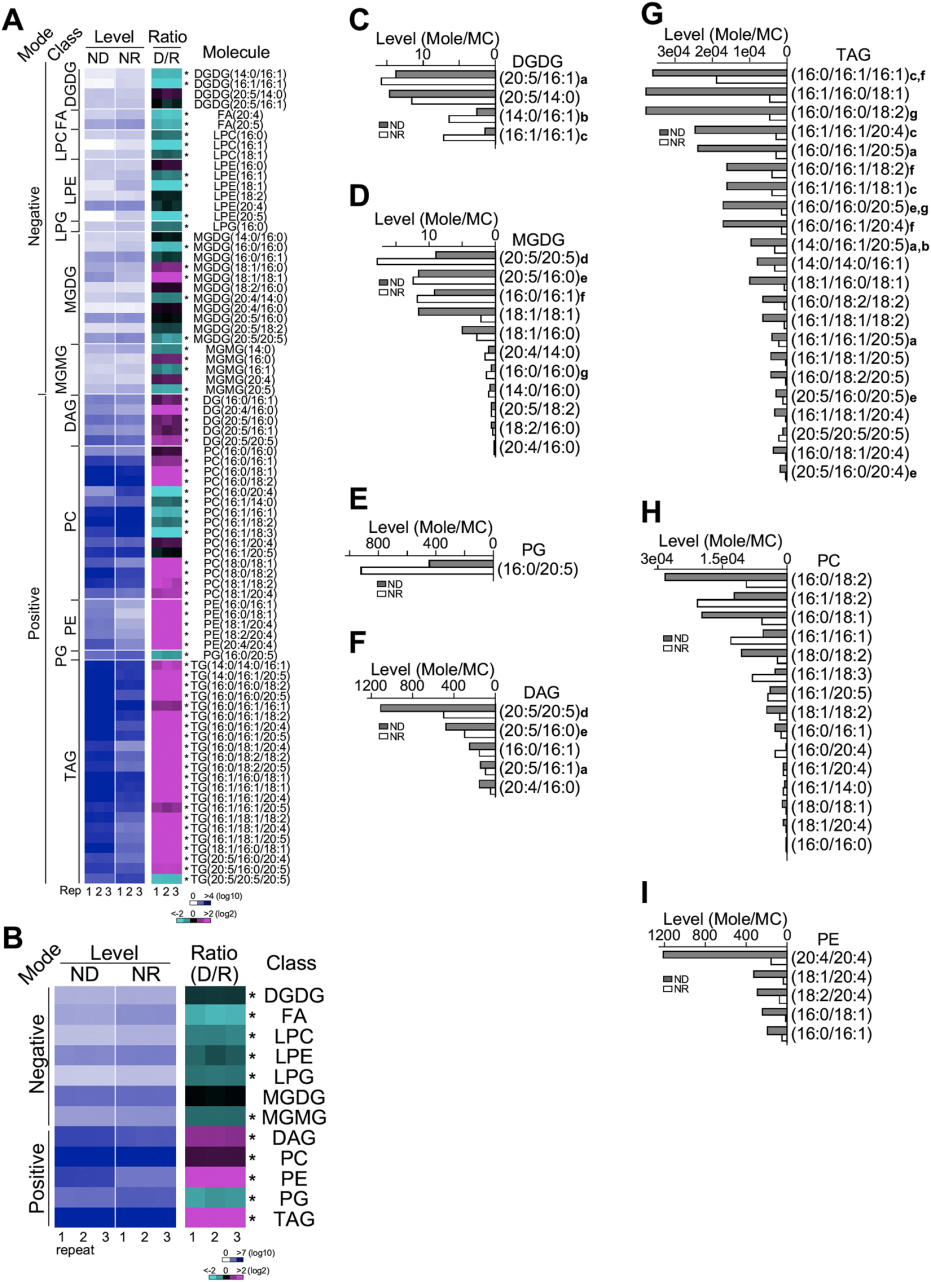
Lipidomic profiling of PJ12 cells under ND and NR conditions. (**A**) Comparison between lipidomic profiles between ND and NR conditions. Heat-map shows the level (mole of molecules based on peak area divided by MC per million cells under ND and NR condition) and ratio (ND level/NR level) of individual lipid molecules. Ion mode (Mode) and lipid class (Class) are also shown. Level and ratio of individual molecules are shown in blue-white (from high to low in log 10 scale) and magenta-black-cyan (from increase to unchanged and to decrease in log 2 scale) color schemes, respectively. Repeated experiments and scale bars are shown at the bottom. Asterisk (*) indicates the significant difference of lipid levels between ND and NR conditions (level change > 1.5-fold, p-value < 0.05). (**B**) Comparison of lipid classes between ND and NR conditions. The display is identical to (**A**). Asterisk (*) indicates significantly difference between ND and NR (level change > 1.2-fold, p-value < 0.05). (**C**) Bar-plot shows the level of DGDG lipids under ND and NR conditions. Photosynthetic membrane lipids (DGDG and MGDG) denoted with alphabetic letter can be traced in neutral lipids (DAG and TAG). Bar-plots (**D–I**) show the level of MGDG, PG, DAG, TAG, PC, and PE lipids under ND and NR conditions, respectively.

By using LC-MS/MS-based lipidomic analysis (see Materials and Methods), we identified a total of 80 lipid species in 12 lipid classes, namely, DGDG (digalactosyldiacylglycerol), FFA (free fatty acid), LPC (lysophosphatidylcholine), LPE (lysophosphatidylethanolamine), LPG (lysophosphatidylglycerol), MGDG (monogalactosyldiacylglycerol), MGMG (monogalactosylmonoacylglycerol), DAG (diacylglycerol), PC (phosphatidylcholine), PE (phosphatidylethanolamine), PG (phosphatidylglycerol), and TAG (triacylglycerol) in PJ12 cells under ND and NR conditions in triplicate (Fig. 3A, Supplemental Table S2). While levels of four lipid classes (DAG, PC, PE, and TAG) under ND condition were increased (level change > 20%, p-value < 0.05) compared to NR condition based on lipid levels per million cells, levels of seven lipid classes (DGDG, FA, LPC, LPE, LPG, MGMG, and PG) were decreased (level change > 20%, p-value < 0.05) (Fig. 3B). This result implied that levels of storage lipid TAG and its precursor DAG and editing lipid pool of PC and PE appeared to increase in response to nitrogen deprivation, consistent with the notion that levels of TAG increases under ND condition^3^. It also indicated that levels of photosynthetic membrane-lipid species in DGDG, MGDG, and PG under ND condition were decreased compared to that of NR condition.

Given that levels of photosynthetic membrane-lipids (such as DGDG and PG and some lipid species in MGDG) and neutral lipids (TAG and DAG) under ND condition were decreased and increased (respectively) compared to NR condition (Fig. 3C–I), we hypothesized that some photosynthetic membrane lipid species in DGDG, MGDG, and PG contributed to the accumulation of neutral lipid TAG and its precursor DAG upon ND. To test this possibility, we searched for major lipid species (all lipid species if a lipid class contained five or less lipid species and top 50% lipid species if a lipid class contained ten or more lipid species) whose levels decreased in lipid classes DGDG, MGDG, and PG but increased in lipid classes TAG and DAG upon nitrogen deprivation. To this end, we found that seven major lipid species whose levels were decreased in DGDG (see species a–c in Fig. 3C) and MGDG (see species d–g in Fig. 3D) but increased in DAG (see a, d, and e in Fig. 3F) and TAG (see a–c and e–g in Fig. 3G). For instance, lipid species a DGDG(20:5/16:1) (at *sn*-1/*sn2* positions of the glycerol backbone) (see a in Fig. 3C) whose lipid signature could be found in DAG(20:5/16:1) (see a in Fig. 3F) and TAG(16:0/16:1/20:5, 14:0/16:1/20:5, and 16:1/16:1/20:5) (*sn*−1 and *sn*3 are symmetric) (see a in Fig. 3G).This result provided the evidence that photosynthetic membrane-lipid species in DGDG and MGDG contributed to the accumulation of neutral lipids DAG and TAG in PJ12 cells upon ND. We found that level of PG(16:0/20:5) species was decreased upon ND (see Fig. 3E), but its lipid signature was not found in other lipid classes, suggesting that the lipid underwent editing or recycling. We found that most of the major lipid species in DGDG, MGDG, DAG, and TAG were derived from prokaryotic synthetic pathway (i.e., C16:X at *sn2* position). However, major lipid species in PC and PE hardly showed any C16:X at *sn2* position (Fig. 3H,I). We found that some major PC lipid species whose levels were increased and some were decreased, while levels of all PE lipid species were increased. These results were consistent with the notion that PC and PE played an important role in lipid editing, providing a pool of elongated and unsaturated lipids^29^.

### RNA-seq-based *de novo* assembly of PJ12 transcriptome

To investigate the alteration of genes involved in metabolisms of lipid and its precursors under ND and NR conditions, we performed de novo transcriptomic analysis of cells three days after medium shift to ND and NR conditions in triplicate (see Materials and Methods). All samples were subjected to RNA-seq analysis using BGISEQ-500 platform with PE100 scheme (BGI, Shenzhen, China). The resulting sequences were pooled for de novo assembly using Trinity^39^ (see Materials and Methods). To this end, a total of 12,609 high quality EST (non-redundant, annotated, no single missing data in triplicate samples under both conditions) (Supplemental Table S1) were obtained. Distribution of EST length and abundance and EST’s top 10 most associated GO functions and KEGG pathways were shown in Supplemental Fig. S1.

We found five ESTs encoding light-harvesting function in the top 10 most abundant ESTs based on normalized level TPM (transcripts per million) in cells under NR condition, which was 175-fold enrichment (p-value = 4.72e-11) compared to background (36 in 12,609 ESTs). On the other hand, no light-harvesting but 3 ribosomal proteins-encoding ESTs were found in the top 10 most abundant ESTs under ND condition, exhibiting a 13.3-fold enrichment (p-value = 0.0012) of ribosomal proteins compared to background (284 in 12,609 ESTs). Comparison to the NR condition, decreased transcriptional level of photosynthetic genes may suggest a decreased photosynthetic activity under ND condition. This was consistent with the observation that maximum PSII quantum yield was decreased upon ND stress (see Fig. 1D). High transcriptional level of genes involved in protein synthesis under ND condition would imply an increased protein turnover rate for synthesis of new proteins.

**Figure 4:**
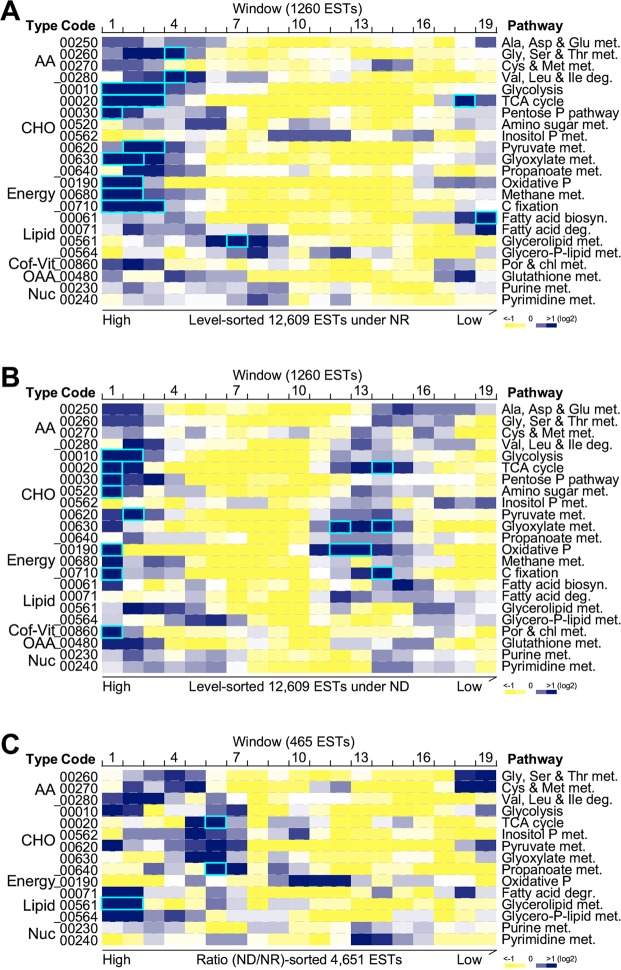
Pathway-based functional analysis of transcriptional profiles under NR and ND conditions. (**A**) Enrichment of pathway-associated ESTs at various levels based on rank under NR condition. Heat-map showing the occurrence frequency of ESTs associated with various metabolic pathways indicated. Blue-white-yellow color key is indicated as the ratio of occurrence increased, unchanged, and decreased at the bottom. Function enriched windows are highlighted with cyan-colored rectangles. (**B**) Enrichment of pathway-associated ESTs at various levels based on rank under ND condition. The display is identical to (**A**). (**C**) Enrichment of pathway-associated ESTs at various ratio (ND/NR) based on rank. The display is identical to (**A**).

To investigate enrichment of ESTs involved in various metabolic pathways occurred within a certain transcription level, we performed sliding window analysis (see Materials and Methods). In this analysis, a window of 1,260 ESTs in size (i.e., 10% of total ESTs tested) were moved along the sorted ESTs based on rank by level (TPM) with a moving step of 630 ESTs (i.e., 50% of the window size). A total of 19 windows were recorded (pathways that were associated ESTs of 60 or more were considered). Occurrence of ESTs associated with a KEGG pathway in a window was compared to background. To this end, we found that five carbohydrate and three energy metabolic pathways exhibited increased occurrence (frequency > 2-fold, p-value < 0.05 after Bonferroni modification) in the top three most abundant windows under NR condition (Fig. 4A). On the other hand, five out of eight metabolic pathways enriched in the top three most abundant EST windows under NR condition were also found to be over represented in the top most abundant windows under ND condition (Fig. 4B). However, no common metabolic pathways enriched in the medium or low abundant EST windows were found under ND and NR conditions.

Of a total of 12,609 ESTs, 4,651 (37.9%) exhibited differential change (level change > 4-fold, p-value < 0.05) of transcription levels between ND and NR conditions, indicating a high impact on transcriptional machinery in cells upon nitrogen deprivation. By using the sliding window to sorted ESTs based on rank by ratio (ND/NR) (pathways that were associated with ESTs of 25 or more were considered), we found that glycerolipid metabolism was enriched in the top three windows, consistent with the increase of TAG level in ND cells (Fig. 4C).

### Transcription levels of enzymes involved in TCA cycle but not Calvin cycle or glycolysis are upregulated upon nitrogen deprivation in PJ12

**Figure 5:**
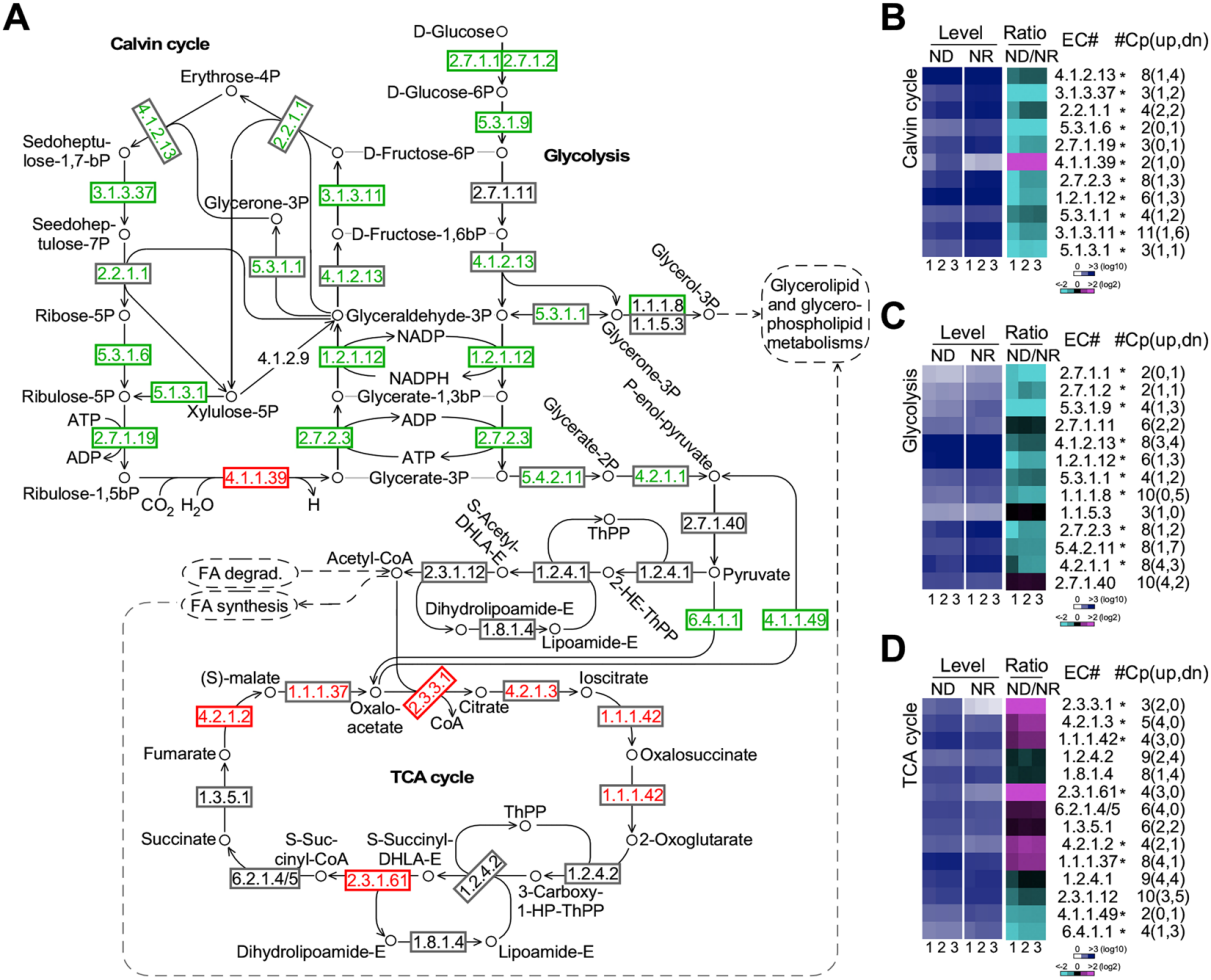
Differential transcriptions of enzymes involved in Calvin cycle, glycolysis, TCA cycle under ND and NR conditions. (**A**) Schematic pathways showing Calvin cycle, glycolysis, TCA cycle. EC number of enzymes and metabolites are indicated. Transcriptionally increased and decreased enzymes based on the sum of isoforms are indicated in red and green, respectively. Enzymes without significant change are indicated in black. Enzymes not found in this analysis are shown without box. (**B**) Transcription profiles of enzymes involved in Calvin cycle. Heat-maps indicate transcription levels of enzymes (sum of all copies of genes if exists) involved in Calvin cycle under ND and NR conditions (blue-white scheme) and ratio of ND/NR (magenta-black-cyan scheme). EC number (EC#) is shown. Asterisk (*) indicates the significant change (level change > 1.5-fold, p-value < 0.05) based on the sum of all isoform levels. Total copy number of genes (#Cp) and number of upregulated (up) and downregulated (dn) individual genes in parentheses are shown. Repeat experiments and color keys are indicated at the bottom. (**C**,**D**) Transcription profiles of enzymes involved in glycolysis and TCA cycle, respectively. The display is identical to (**B**).

We investigated the alteration of transcription levels of genes involved in Calvin cycle, glycolysis, and TCA cycle based on the sum of isoform levels of individual enzymes when available (Fig. 5A). All enzymes were indicated with Enzyme Commission (EC) numbers whose names were listed in Table 1. We found that each EC number was associated with 4.37 copies of genes/ESTs in average based on the PJ12 transcriptome (in this study), which was similar to that of *Chlamydomonas reinhardtii* (5.89 copies of genes in average based on the genome)^40^, *Chlorella variabilis* (4.66 copies of genes in average)^41^, and *Dunaliella salina* (5.24 copies of genes/ESTs in average based on the transcriptome)^42^. All redundant copies of enzymes in PJ12 were found to be originated from paralogous genes (encoding identical proteins or isoforms)^43^ except for less than 3% ESTs that were derived from alternatively-spliced transcription^44^. We found that transcription levels (sum of multi-copies of genes when present) of ten out of the eleven enzymes in the Calvin cycle under ND condition were downregulated (level change > 1.5-fold, p-value < 0.05) compared to that of NR condition, except rubisco (EC 4.1.1.39) that was upregulated (Fig. 5B, Supplemental Table S3). We noted that all enzymes involved in Calvin cycle possessed multiple copies of paralogous genes except for rubisco (EC 4.1.1.39) that was a single copy gene with two predicted alternatively-spliced transcription isoforms, eight out of which exhibited more downregulated copies (individual gene transcription level change > 1.5-fold, p-value < 0.05) under ND condition than that of NR condition. Downregulation of Calvin cycle activity is consistent with the observation that maximal PSII quantum yield (Fv/Fm) under ND condition was reduced compared to that of NR condition (see Fig. 1D).

**Table 1:** List of EC number and name of enzymes used in this study.

EC#	Description
1.1.1.100	3-oxoacyl-[acyl-carrier protein] reductase
1.1.1.35	3-hydroxyacyl-CoA dehydrogenase
1.1.1.37	malate dehydrogenase
1.1.1.42	isocitrate dehydrogenase
1.1.1.8	glycerol-3-phosphate dehydrogenase (NAD^+^)
1.1.5.3	glycerol-3-phosphate dehydrogenase (FAD)
1.11.1.6	catalase
1.14.19.1	stearoyl-CoA desaturase (Delta-9 desaturase)
1.14.19.2	acyl-[acyl-carrier-protein] desaturase
1.14.19.23|1.14.19.45	acyl-lipid omega-6 desaturase (Delta-12 desaturase)
1.14.19.25|1.14.19.35	glycerolipid omega-3-fatty acid desaturase
1.14.19.3	fatty acid desaturase; Linoleate desaturase
1.2.1.12	glyceraldehyde 3-phosphate dehydrogenase
1.2.4.1	pyruvate dehydrogenase E1 component subunit
1.2.4.2	2-oxoglutarate dehydrogenase E1 component
1.3.1.9|1.3.1.10	enoyl-[acyl-carrier protein] reductase
1.3.3.6	acyl-CoA oxidase
1.3.5.1	succinate dehydrogenase (ubiquinone)
1.8.1.4	dihydrolipoamide dehydrogenase
2.2.1.1	transketolase
2.3.1.12	pyruvate dehydrogenase E2 component
2.3.1.15	glycerol-3-phosphate O-acyltransferase
2.3.1.158	phospholipid: diacylglycerol acyltransferase
2.3.1.16	acetyl-CoA acyltransferase
2.3.1.180	3-oxoacyl-[acyl-carrier-protein] synthase III
2.3.1.199	fatty acid elongase
2.3.1.20	diacylglycerol O-acyltransferase
2.3.1.22	2-acylglycerol O-acyltransferase
2.3.1.25|2.3.1.23	lysophosphatidylcholine acyltransferase
2.3.1.39	[acyl-carrier-protein] S-malonyltransferase
2.3.1.41|2.3.1.179	3-oxoacyl-[acyl-carrier-protein] synthase II
2.3.1.51	1-acyl-sn-glycerol-3-phosphate acyltransferase
2.3.1.61	2-oxoglutarate dehydrogenase E2 component
2.3.3.1	citrate synthase
2.4.1.241	digalactosyldiacylglycerol synthase
2.4.1.46	1,2-diacylglycerol 3-beta-galactosyltransferase
2.7.1.1	hexokinase
2.7.1.11	6-phosphofructokinase
2.7.1.19	phosphoribulokinase
2.7.1.2	glucokinase
2.7.1.40	pyruvate kinase
2.7.2.3	phosphoglycerate kinase
2.7.7.14	ethanolamine-phosphate cytidylyltransferase
2.7.7.15	choline-phosphate cytidylyltransferase
2.7.7.41	phosphatidate cytidylyltransferase
2.7.8.1	ethanolaminephosphotransferase
2.7.8.2	choline/ethanolamine phosphotransferase
2.7.8.5	CDP-diacylglycerol–glycerol-3-phosphate 3- phosphatidyltransferase
3.1.1.26	galactolipase
3.1.1.3	triacylglycerol lipase
3.1.1.4	phospholipase
3.1.3.11	fructose-1,6-bisphosphatase
3.1.3.37	sedoheptulose-bisphosphatase
3.1.3.4	phosphatidate phosphatase
4.1.1.39	ribulose-bisphosphate carboxylase small chain
4.1.1.49	phosphoenolpyruvate carboxykinase (ATP)
4.1.2.13	fructose-bisphosphate aldolase
4.2.1.1	carbonic anhydrase
4.2.1.17	enoyl-CoA hydratase
4.2.1.2	fumarate hydratase
4.2.1.3	aconitate hydratase
4.2.1.59	3-hydroxyacyl-[acyl-carrier-protein] dehydratase
5.1.3.1	ribulose-phosphate 3-epimerase
5.3.1.1	triosephosphate isomerase
5.3.1.6	ribose 5-phosphate isomerase
5.3.1.9	glucose-6-phosphate isomerase
5.4.2.11	2,3-bisphosphoglycerate-dependent phosphoglycerate mutase
6.2.1.3	long-chain acyl-CoA synthetase
6.2.1.4|6.2.1.5	succinyl-CoA synthetase subunit
6.4.1.1	pyruvate carboxylase
6.4.1.2	acetyl-CoA carboxylase carboxyl transferase subunit

Similarly, ten out of 13 enzymes involved in glycolysis under ND condition exhibited significant downregulation (level change > 1.5-fold, p-value < 0.05) compared to that of NR condition (Fig. 5C, Supplemental Table S4). Again, all enzymes involved in glycolysis were found to be encoded by multi-copy genes, nine out of which exhibited more downregulated copies (individual gene transcription level change > 1.5-fold, p-value < 0.05) under ND condition than that of NR condition. Glycolysis is known to occur in both the plastid and cytosol. However, we were unable to unambiguously predict all isoforms to specific subcellular localization using various algorithms^24–28^. Given that cells were grown under autotroph condition with decreased photosynthetic sugar production upon ND, one would expect the overall reduction of glycolysis activity in the plastid and cytosol.

Consistent with the reduced Calvin cycle and glycolysis activities, two enzymes (EC 6.4.1.1 and EC 4.1.1.49) that linked triose to TCA cycle were downregulated (level change > 1.5-fold, p-value < 0.05) based on the sum of multi-copies of genes. Six out of the twelve enzymes involved in the TCA cycle were upregulated, of which, seven showed more upregulated copies (individual gene transcription level change > 1.5-fold, p-value < 0.05) under ND condition than that of NR condition (Fig. 5D, Supplemental Table S5). These results suggested that molecules generated from pathways other than Calvin cycle and glycolysis were oxidized in TCA cycle for energy production under ND condition in PJ12 cells.

### Transcription levels of enzymes involved in lipid synthesis but not breakdown are downregulated in response to nitrogen deprivation in PJ12

**Figure 6:**
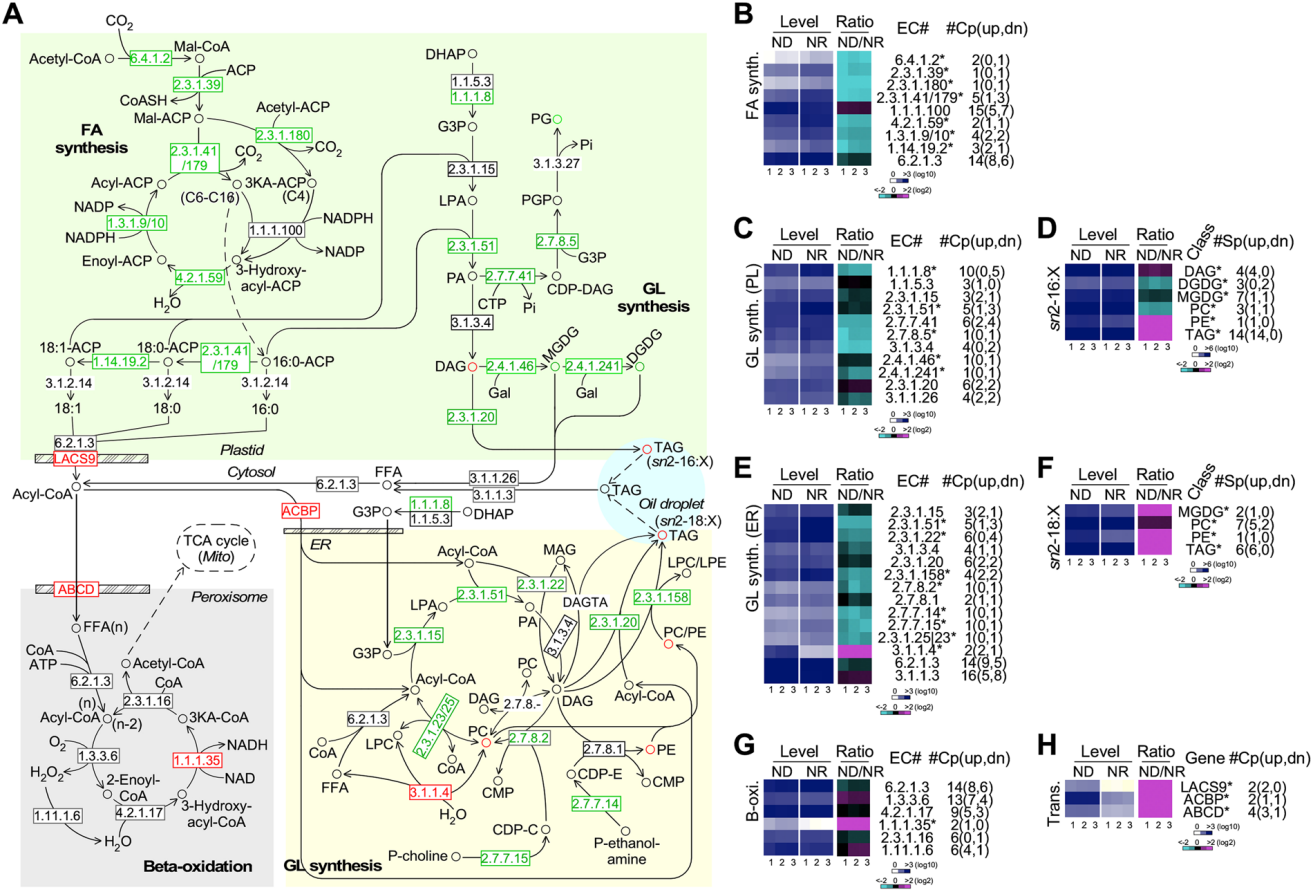
Differential transcriptions of enzymes involved in fatty acid, photosynthetic membrane lipid, and triacylglycerol synthesis and beta-oxidation under ND and NR conditions. (**A**) Schematic pathways showing fatty acid (FA) synthesis, glycerolipid (GL) synthesis in the plastid (PL) and in the ER, beta-oxidation, and acyl-CoA transport. Metabolite whose level increased and decreased are shown in red and green, respectively. The display is identical to Fig. 3 (**A**). (**B**,**C**) Transcription profiles of enzymes involved in fatty acid (FA) synthesis and glycerolipid (GL) synthesis in the plastid (PL). The display is identical to Fig. 5B. (**D**) Profiles of “prokaryotic” pathway-synthesized lipids. Heat-maps indicate levels of lipid class containing C16:X at sn2 position of the glycerol backbone under ND and NR conditions (blue-white scheme) and ratio of ND/NR (magenta-black-cyan scheme). Lipid class (Class) is shown. Asterisk (*) indicates the significant level change (level change > 1.25-fold, p-value < 0.05) based on the sum of all lipid species in a lipid class. Total number of lipid species (#Sp) and number of upregulated (up) and downregulated (dn) individual species in parentheses are shown. Repeat experiments and color keys are indicated at the bottom. (**E**) Transcription profiles of enzymes involved in glycerolipid (GL) synthesis in the ER. The display is identical to 5B. (**F**) Profiles of “eukaryotic” pathway-synthesized lipids. The display is identical to (**D**). (**G**,**H**) Transcription profiles of enzymes involved in β-oxidation and acyl-CoA transport. The display is identical to Fig. 5B.

Subsequently, we investigated the change of transcription levels of genes involved in fatty acid synthesis, glycerolipid synthesis in the plastid and in endoplasmic reticulum (ER), and β-oxidation (Fig. 6A). We found that seven out of nine enzymes in the fatty acid synthetic pathway based on the sum of multi-copy genes transcriptional levels when available under ND condition were significantly downregulated (level change > 1.5-fold, p-value < 0.05) compared to that of NR condition, including two single copy enzymes (EC 2.3.1.39 and EC 2.3.1.180) (Fig. 6B, Supplemental Table S6).

Based on the sum level of all copies of genes when available, we found that five out of eleven enzymes involved in glycerolipid synthesis in the plastid under ND condition were downregulated (level change > 1.5-fold, p-value < 0.05) when compared to that of NR condition (Fig. 6C, Supplemental Table S7). At this point, we were unable to distinguish the subcellular localization of individual enzyme isoforms. Nevertheless, three out of five downregulated enzymes were single copy genes or exclusively localized in the plastid, indicating that the overall reduction of glycerolipid synthesis occurred upon ND in PJ12 cells. Based on the lipidomic profile of “prokaryotic” lipids (16:X at the *sn*2 position of the glycerol backbone), we found that levels of DGDG and MGDG were decreased (level change > 1.25-fold, p-value < 0.05), consistent with the downregulation of single copy enzymes EC 2.4.1.46 and EC 2.4.1.241 (Fig. 6D, Supplemental Table S8). However, while enzyme EC 2.3.1.20 was downregulated based on the sum of all copies of gene transcription levels, the TAG content was increased upon ND. This discrepancy could be resolve when subcellular localization of isoforms was available. We noted that, of the six copies of genes encoding for EC 2.3.1.20, two each were upregulated, unchanged, and downregulated. We proposed that the upregulated isoforms were responsible for accumulation of TAG derived from prokaryotic pathway upon ND.

Subsequently, we investigated “eukaryotic” pathway of the glycerolipid synthesis in the ER. We found that, of 14 enzymes involved in the “eukaryotic” pathway, seven were downregulated (level chnge > 1.5-fold, p-value < 0.05) based on sum of all copies of gene levels when available, four of which were single copy enzymes on ER (Fig. 6E, Supplemental Table S9). We found that TAG synthesis enzymes EC 2.3.1.20 and EC 2.3.1.158 with multiple isoforms were downregulated based on the sum of all copies of gene levels. However, lipidomic profile indicated that levels of “eukaryotic” neutral lipid TAG and phospholipids PC and PE under ND condition were increased (level change > 1.2-fold, p-value < 0.05) compared to that of NR condition (Fig. 6F, Supplemental Table S10). Hence, it was possible that upregulated isoforms of EC 2.3.1.20 and EC 2.3.1.158 would be responsible for the TAG increase. Although the overall level of MGDG appeared to be unchanged (see Fig. 3B), it was found that levels of MGDG synthesized through the “prokaryotic pathway” and “eukaryotic pathway” were decreased and increased, respectively (see Fig. 6D,F).

We found that none of the six enzymes involved in β-oxidation under ND condition was downregulated (level change > 1.5-fold, p-value < 0.05) compared to that of NR condition (Fig. 6G, Supplemental Table S11). In fact, one enzyme in β-oxidation was even significantly upregulated (level change > 1.5-fold, p-value < 0.05) upon ND. Additionally, we found that transcription levels of transporter LACS9 and ABCD and acyl-CoA-binding protein ACBP under ND condition were upregulated (level change > 2-fold, p-value < 0.05) (Fig. 6H, Supplemental Table S12).

### Transcription level of enzymes involved in polyunsaturated fatty acid (PUFA) synthesis upon ND are downregulated

**Figure 7:**
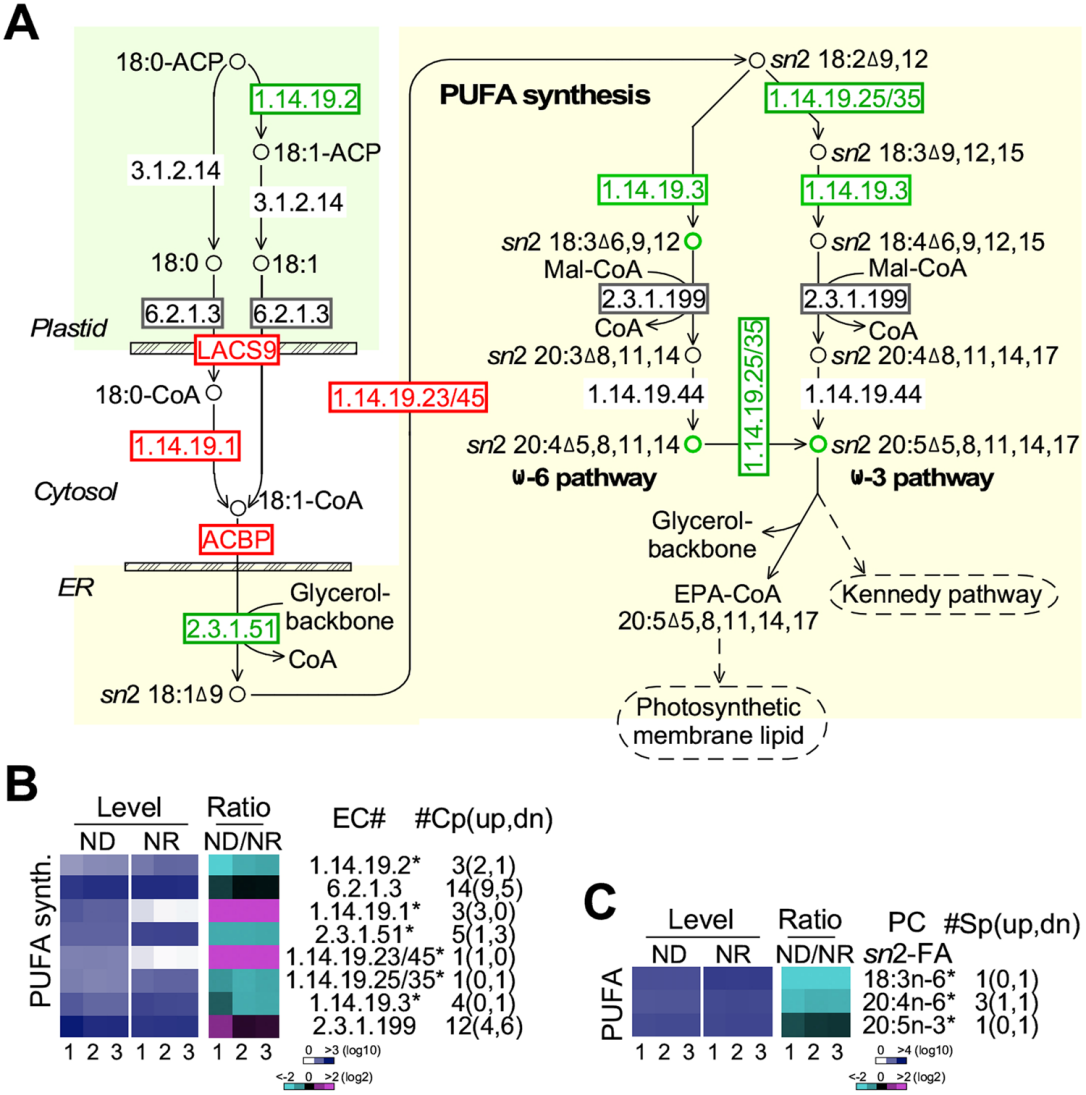
Differential transcriptions of enzymes involved in polyunsaturated fatty acid (PUFA) synthesis under ND and NR conditions. (**A**) Schematic pathways showing polyunsaturated fatty acid (FA) synthesis. The display is identical to Fig. 6A. (**B**) Transcription profiles of enzymes involved in polyunsaturated fatty acid (FA) synthesis. The display is identical to Fig. 5B. (**C**) Level changes of PUFA in PC at the *sn*2 position of the glycerol backbone. The display is identical to Fig. 6D.

We investigated change of enzyme transcript levels and metabolite levels associated with PUFA synthesis in PJ12 cells under ND and NR conditions (Fig. 7A). Based on the sum of multi-copy gene transcription levels when available, we found that two and four out of eight enzymes under ND condition were upregulated and downregulated (level change > 1.5, p-value < 0.05) compared to that of NR condition, respectively (Fig. 7B, Supplemental Table S13). Three out of four enzymes (EC 2.3.1.51, EC 1.14.19.25/35, and EC 1.1.4.19.3) involved in *sn2* linoleic acid desaturation and elongation under ND condition were downregulated (level change > 1.5-fold, p-value < 0.05), except EC 1.14.19.23/45.

PC and PE are major lipids involved in lipid elongation and desaturation^45^. Based on our lipidomic profiles, levels of PC were at least one magnitude higher than that of PE. Hence, we focused on the analysis of level changes of *sn*2 PUFA in PC lipids. Of 15 PC lipid species, one each contained 18:3n-6 and 20:5n-3 and three contained 20:4n-6 at *sn2* position. Levels of PUFA at *sn*2 position in PC lipid class under ND condition were found to be decreased (level change > 1.25-fold, p-value < 0.05) compared to that of NR (Fig. 7C, Supplemental Table S14), consistent with the transcriptional analysis.

## Discussion

*Nannochloropsis* spp. are considered as promising strains for biofuel and biomass production^3,46^. In this study, we show that the regulation of genes involved in TAG synthesis in *Nannochloropsis* sp. PJ12 cells under ND condition. rDNA sequence-based phylogenetic analysis shows that PJ12 is most closely related to *N*. *oceanica* (see Fig. 1C). Comparative analysis of homologous sequences (e-value < 1e-07) between the PJ12 transcriptome and the previously reported genomes of *Nannochloropsis* spp. indicates that PJ12 shares homologous sequences with 94.4% (11,341 out of a total of 12,012 genes in the genome), 92.3% (10,271 out of 11,129 genes), 82.1% (7,433 out of 9,053 genes), and 40.4% (8,863 out of 21,957 genes) of the *N*. *oceanica* CCMP1779^8^, *N*. *oceanica* LAMB0001^9^, *N*. *gaditana* CCMP526^47^, and *N*. *gaditana* B-31^11^ genomes, suggesting that PJ12 is most closely related to *N*. *oceanica* CCMP1779 (see Supplemental Method S1).

In this analysis, we show that the top 3 most abundant acyl lipids as FAMEs are C16:0, C16:1, and C20:5 in PJ12 (see Fig. 2D). While levels of C16:0 and C16:1 are increased 3 days after ND, C20:5 level is decreased. In a study by Meng *et al*.^35^, they have reported the same observation in *N*. *oceanica*. These are in clear contrast with the fatty acid profiles of *C*. *reinhardtii* CC125^48^ and CC4326^49^, whose major fatty acid are C18:3, C16:0 and C16:4 without C20:5. But they have the same behavior that the saturated fatty acid C16:0 and the monounsaturated fatty acid C16:1 are increased with the polyunsaturated fatty acids C16:4, C18:3 and C20:5 decreased in the nitrogen-starved cells compare to the nitrogen-replete cells.

Based on the transcriptomic profiling analysis of PJ12, we show that, while Calvin cycle and glycolysis pathway were transcriptionally downregulated under ND condition, TCA cycle activity is upregulated (see Fig. 5). Decreased quantum yield (see Fig. 1D) reduces photosynthetic sugar flux into TCA cycle for energy production under ND condition. Hence, elevated transcription level of genes involved in TCA cycle activity would suggest that molecules to be oxidized through TCA cycle are likely to be generated from pathways other than Calvin cycle and glycolysis. Li *et al*.^23^ have shown that transcription of enzymes involved in Calvin cycle and TCA cycle are downregulated and upregulated, respectively, upon ND in IMET1. Although based on subcellular localization of enzymes involved in glycolysis is downregulated in plastid and upregulated in cytosol, the sum of transcription levels of all enzyme in plastid and cytosol appears to be downregulated^23^. Hence, transcriptional change upon ND in IMET1 is similar to that in PJ12. On the other hand, in *C*. *reinhardtii*, Carpinelli *et al*.^11^ have shown that transcriptional levels of enzymes involved in TCA cycles is hardly altered. However, enzymes in glycolysis is overall decreased, though its cytosol isoforms are unchanged but plastid isoforms are downregulated.

In this study, we show that cell mass density decreases, whereas cell volume increases upon ND stress (see Fig. 2A,C). This is accompanied with the shrunk of chloroplast (see Fig. 2B)^50^. It has been shown that the density of thylakoid membrane is higher than the cell mass density^49,51^. Therefore, we propose that cell mass density decrease under ND condition is attributed to both increase of low-density neutral lipids and decrease high-density photosynthetic membrane lipids.

Using LC-MS/MS-mediated lipidomic analysis, we show that levels of DGDG and PG are decreased, while PC, PE, and TAG levels are increased. Though the overall level of MGDG remains unchanged, levels of MGDG synthesized through the “prokaryotic” and “eukaryotic” pathways are decreased and increased, respectively (see Figs 3B and 6D,F). Meng *et al*.^35^ have showed that levels of MGDG, DGDG, PC, PE, and PG are all decrease 3 days after ND, but TAG level is increased in an unknown *N*. *oceanica* isolate. On the other hand, in a study by Li *et al*.^23^, they have showed that levels of MGDG, DGDG, PC, and PG are decreased 3 days after ND, while levels of TAG and PE are increased in *N*. *oceanica* IMET1. Although the level change of some lipid classes in various *N*. *oceanica* isolates appear to vary upon ND, levels of photosynthetic membrane lipids such as MGDG, DGDG, and PG synthesized via “prokaryotic” pathway are always decreased, while the level of TAG is found to be increased in these *N*. *oceanica* isolates. Significantly, a similar observation in *C*. *reinhardtii* has been reported^52^. The authors have shown that levels of MGDG, DGDG, and PG are reduced, while TAG level is increased 2 days after ND in *C*. *reinhardtii* CC-400.

Based on transcriptomic profiling, we show that enzymes involved in de novo fatty acid synthesis, “prokaryotic” (in the plastid) and “eukaryotic” (in the ER) glycerolipid synthesis are, in general, downregulated in PJ12 (see Fig. 6). Similarly, Li *et al*.^23^ have shown that transcriptional level of the FASII is downregulated upon ND in IMET1. In the contrary, genes involved in FASII in *C*. *reinhardtii* have been shown to be unaffected upon ND^11^. Downregulation of glycerolipid synthesized via “prokaryotic” pathway is consistent with the observation of the shrunk chloroplast. On the other hand, based on the lipidomic profiling, levels of photosynthetic membrane glycerolipids such as DGDG and MGDG synthesized via “prokaryotic” pathway are decreased (see Fig. 6D), in agreement with the transcriptional profiling.

Plastidic and cytoplasmic membrane lipids may undergo hydrolysis through β-oxidation to produce acetyl-CoA or convert into TAG as storage molecule. However, enzymes involved in membrane lipids disassembly are less well-defined, except for converting DAG into TAG. Multiple pathways converting DAG to TAG are documented^53^. Diacylglycerol transacylase (DAGTA) transfers an acyl molecule from one DAG to the other, producing a MAG and a TAG (see Fig. 6A)^53^. On the other hand, DAG can be converted into TAG in an acyl-CoA-dependent manner by acyl-CoA:diacylglycerol acyltransferase (DGAT, EC 2.3.1.20) or in an acyl-independent manner by phospholipid:diacylglycerol acyltransferase (PDAT, 2.3.1.158). Both enzymes are found to contain multiple isoforms in PJ12, some of which are upregulated and others are downregulated (see Fig. 6E). In *N*. *oceanica* IMET1, Li *et al*.^23^ have shown a similar scenario for DGAT, but only one downregulated copy of PDAT. Carpinelli *et al*.^11^ have shown that, in *C*. *reinhardtii*, genes involved in TAG biosynthesis stay unaltered upon ND.

Genes possessing multiple copy numbers are a common phenomenon in algae and plant^32,42–44^. In this study, we show that increase or decrease of metabolite flux in various pathways can be predicted based on the sum of transcription levels of multi-copy enzyme-encoding genes (see Figs 5–7). However, discrepancy occurs in prediction of increased or decreased flux from DAG to TAG upon ND. One may resolve this issue by predicting subcellular localization of the enzyme isoforms^23,31^. However, no single algorithm can unambiguously predict subcellular localization of all isoforms^24–28,30^. In this case, lipidomic profiling is the right method to resolve this issue unambiguously.

Based on lipidomic profiling, we show that the flux from DAG to TAG by both “prokaryotic” and “eukaryotic” pathways is increased upon ND (see Fig. 6D,F), majority (~70%) of which are generated through the “prokaryotic” pathway based on the C16:X at the *sn*2 position of the glycerol backbone.

Membrane-bound fatty acid desaturases (e.g., EC 1.14.19.23 and EC 1.14.19.25) are known to preferentially use substrate like PC^54^. We show, in this study, that EPA level is reduced upon ND (see Fig. 2D). Our transcriptomic profiling indicates that 3 out of 5 enzymes involved in PUFA synthesis pathways on ER are downregulated (see Fig. 7A,B). Meanwhile, the lipidomic profiling shows that levels of PC with C18:3n-6, C20:4n-6, and C20:5n-3 at the *sn*2 position of the glycerol backbone are downregulated (see Fig. 7C). These results are consistent with the result of GC-MS analysis (see Fig. 2D) and further extend that EPA is synthesized through the omega-6 pathway in PJ12, similar to the observation that 20:4n-6 but not 20:4n-3 is a preferable precursor for 20:5n-3 production in microalga *Porphyridium cruentum*^55^. PC is also a key molecule subjected to lipid editing^56^, which is responsible for incorporation of elongated and unsaturated acyl molecules into DAG and TAG^57^. This may explain that, unlike other lipid classes, levels of major PC species display non-uniform change under ND condition (see Fig. 3H).

Long chain polyunsaturated fatty acids (PUFA) are a major component in photosynthetic membrane^58^. Consistent with this, we show that 20:5n-3 is present in the top most abundant lipid species of classes DGDG and MGDG (see Fig. 3C,D). Levels of these photosynthetic membrane lipids decrease under ND condition, which undergoes β-oxidation in peroxisome exclusively in plant^59^. β-oxidation in peroxisome produces hydrogen peroxide (H_2_O_2_) that is toxic to cell. Hence, it is necessary to express catalase (EC 1.11.1.6) to convert H_2_O_2_ to H_2_O. In this study, we show the detection of catalase transcription, which would suggest that, similar to plant, the fatty acids in PJ12 are hydrolyzed in peroxisome upon ND (see Fig. 6G).

The product of acetyl-CoA through β-oxidation can either be used for de novo fatty acid synthesis or further oxidization via TCA cycle. Based on the downregulation of Calvin cycle, glycolysis, and fatty acid synthesis and upregulation of TCA cycle and β-oxidation, we propose that acetyl-CoA generated from β-oxidation is most likely to be channeled to TCA cycle for energy production in PJ12 under ND condition.

Galactolipids are a major component of photosynthetic membrane-lipids^60^. It contains high-level of long-chain unsaturated fatty acids (see Fig. 3C,D)^60^. Processing of fatty acids such as elongation and desaturation primarily occur in the ER. Hence, de novo synthesized fatty acids in the plastid need to be first transported to ER and then returned to plastid after modification^29^. In this study, we show that, while transcriptional levels of genes involved in de novo fatty acid and photosynthetic membrane-lipid synthesis are reduced under ND condition, transcriptional levels of acyl-CoA transport activities between organelles are increased (see Fig. 6). This result suggests that increased acyl-lipid transport activity is likely to be a result of elevated acyl-CoA transportation from plastid to peroxisome for degradation and to ER for modification.

It is known that cell growth rate is negatively correlated with the lipid accumulation rate^61^. Consistent with this, we find that ND induces TAG accumulation in PJ12, but suppresses cell mass increase (see Fig. 1D). For maximum yield of cell mass and lipids, cells should be first allowed growing under NR condition to reach the maximum cell density without apparent decrease of growth rate, e.g., at day 10 for ND treatment (see Fig. 1A). This would increase the starting cell mass for ND treatment by nearly two-fold when compared to medium shift at day 6. Next, cells are subjected to ND for TAG accumulation. In this case, after ND for 3 days, TAG content would be increased by 5-fold compared to that under NR condition (Figs 2E and 3B). With the similar TAG content but twice amount of the cell mass for samples shifting medium to ND at day 10 when compared to day 6, total TAG yield would increase by 2-fold. This two-phase growth scheme would be optimal for efficient production of cell mass and lipids.

## Conclusion

Based on transcriptomic profiling, we show that Calvin cycle and glycolysis activities are downregulated upon ND stress in PJ12. In contrast, TCA cycle activity is upregulated. Furthermore, activities of de novo fatty acid synthesis and glycerolipid synthesis in the plastid and ER are downregulated under ND condition compared to that of NR condition. However, β-oxidation activity is not downregulated. According to lipidomic profiling, level of photosynthetic lipids DGDG and PG are reduced, while neutral lipids DAG and TAG are increase under ND condition. Taken together, our analysis demonstrate that the neutral lipids are primarily derived from modification of membrane-lipids, majority of which comes from “prokaryotic” pathway while minority is from “eukaryotic” pathway in PJ12 under ND condition when compared to that of NR condition. Given the fact that ND induced TAG accumulation is associated with the downregulation of photosynthetic activity, the two-phase growth methodology would be a useful method for production of biofuels and biomaterials.

## Materials and Methods

### Algal strain isolation, culture manipulation

Microalgae that were cultivated in outdoor pond (15 m long x 5 m width x 40 cm depth) which were used as fish feeds in the area of Pengjing city, Liaoning province, China were collected and purified through serial dilution. One of the purified isolates, namely PJ12, was analyzed in this study. The clonal strain was cultivated in modified f/2 medium (in 1 L, it contains 30 g sea salt, 150 mg NaNO_3_, 10 mg NaH_2_PO_4_·H_2_O, 3.15 mg FeCl_3_·6H_2_O, 4.16 mg Na_2_EDTA·2H_2_O, 10 μg CuSO_4_·5H_2_O, 6 μg Na_2_MoO_4_·2H_2_O, 22 μg ZnSO_4_·7H_2_O, 10 μg CoCl_2_·6H_2_O, and 180 μg MnCl_2_·4H_2_O, 2.5 μg Vitamin B_12_, 2.5 μg biotin, and 0.5 μg thiamine HCl)^37^ in glass flask with orbital shaking at 100 rpm, 25 °C, under continuous illumination of 50 µM photon m^−2^ s^−1^. Cell density was determined gravimetrically using a weighing balance (AG204, Mettler-Toledo Inc., Columbus, OH, USA). after collecting 50–100 ml cells on filter paper and oven-dry overnight. Cells at log-phase were subjected to medium shift to f/2 without nitrate and with nitrate. Cells three days after medium shift were collected for RNA-seq analysis with a PE100 protocol on the BGISEQ-500 machine in BGI (Beijing Genomics Institute, Shenzhen, China) and for Lipidomic analysis with a Thermo Orbitrap Elite mass spectrometer (Thermo Fisher, Waltham, MA, USA) in SSB (Shanghai Sensichip Biotech Co. Ltd., Shanghai, China).

### PCR amplification of 18S rDNA sequences

The 18 S rDNA sequences were amplified by two overlapping PCR fragments using primers (F1: 5′-CCAGTAGTCATACGCTTGTCTCAAAGA-3′, R1: 5′-ATAAATCCAAGAATTTCACCTCTGACA-3′, F2: 5′-CTGAGAGACGGCTACCACAT-3′, and R2: 5′-GTTACGACTTCACCTTCCTCT-3′) and PJ12 genomic DNA as template with a standard PCR condition (initial denaturing at 95 °C for 5 min followed by 30 cycles of 95 °C for 30 s, 54 °C for 30 s, and 72 °C for 90 s, and a final extension at 72 °C for 10 min) on a PCR machine (Bio-Rad Laboratories, Inc., Hercules, California, USA). The PCR fragment was subjected to nucleotide sequencing analysis in BGI (BGI, Shenzhen, China). Full length 18 S rDNA of PJ12 was submitted to NCBI nucleotide sequence database with an accession number of MH444206.1.

### Sequence-based phylogenetic analysis

The PJ12 18 S rDNA sequence (MH444206.1) with other sequences from NCBI nucleotide database was aligned using ClustalX with Blosum matrix and the phylogenetic tree was created using the Bootstrapped Neighbor Joining tree method^38^.

### Sucrose gradient centrifugation

Step-wise sucrose (Sinopharm Chemical Reagent Co., Ltd, Shanghai, China) gradient was prepared using 1 ml of undiluted and diluted sucrose solutions such as 90%, 80%, …, 20%, and 10% from saturated or 100% sucrose solution (1.347 g mL^−1^) from the bottom to top of a centrifuge tube (10 mm × 104 mm, thin-wall polyallomer tube, Beckman Coulter, Inc., Atlanta, USA). One ml sample (~1.0e + 08 cells) was overlaid on top of the gradient and centrifuged at 3,500 g for 30 min at 4 °C (Eppendorf Centrifuge 5810 R, Eppendorf AG, Hamburg, Germany). Sucrose density was determined using a refractometer (Atago R5000, Atago USA Inc., Tokyo, Japan).

### Electron microscopic analysis

Cells were harvested from approx. 15 mL culture under ND and NR conditions and washed with phosphate buffer or PB (in 100 mL, 530 mg of NaH_2_PO_4_·H_2_O, 165 mg of Na_2_HPO_4_·6H_2_O, pH7.0) three times. The washed cells were first fixed in 2.5% glutaraldehyde in PB for at least 4 h. After washing with PB, cells were fixed with 1% OsO4 in PB for 1–2 h and washed three times with PB for 15 min at each step. The fixed cells were dehydrated by a graded series of ethanol (30%, 50%, 70%, 80%, 90% and 95%) for about 15 min at each step. Subsequently, cells were dehydrated by pure alcohol for 20 min. In the end, cells were transferred to absolute acetone for 20 min. Dehydrated cells were embedded in Spurr resin (Ted Pella, Inc., Redding, CA, USA) by mixing to a graded series of acetone and Spurr resin mixture (1:1 and 1:3) for 1 h at room temperature and then to the 100% Spurr resin mixture for overnight. Embedded cells were heated at 70 °C for 9 h prior to ultrathin sectioning using LEICA EM UC7 ultratome (Leica microsystems Ltd., Wetzlar, Germany). The resulting sections were stained by uranyl acetate and alkaline lead for 5 min and 10 min, respectively. Sections were analyzed using the Hitachi H-7650 instrument (Hitachi, Ltd., Japan).

### Measurement of cell size and volume

Diameter of cells under ND and NR conditions was measured using automated cell counter (MultisizerTM3 Coulter Counter, Beckman Coulter, Brea, CA, USA) according to manufacturer’s instruction. Based on the diameter or radius, cell volume was approximated as a sphere: *V* = 4 π*r*^3^/3, where *V* for volume and *r* for radius.

### Chlorophyll fluorescence-based analysis of photosynthetic yield

Maximal photosystem II quantum yield (Fv/Fm) of cells under ND and NR conditions was determined using chlorophyll fluorimeter based on pulse amplitude modulation (PAM) technique (Maxi Imaging-Pam system, Heinz Walz GmbH, Effeltrich, Germany) by following the manufacturer’s instruction.

### Transcriptomic analysis

Cell samples (3 days after medium shift to ND and NR media) in quadruplicate (four repeated samples were used in assembly and three of the four repeats were used in profiling analysis) were subjected to RNA-seq analysis with a PE100 protocol using BGISEQ-500 (BGI, Shenzhen, China). A total of 24 Gigabase sequence data (3 Gigabases per sample) or 240 Million reads with 100 bps in length were pooled for de novo assembly of the PJ12 transcriptome. All reads were first subjected to FastQC and Trimmomatic^62^ analyses to remove low-quality sequences including 12 nucleotides at 5-end of each read. The resulting reads with minimal length of 50 nts were subjected to assembly using Trinity^39^. A set of 59,571 non-redundant ESTs were obtained after removal of redundant ESTs using CD-Hit^63^ at a cut-off of 90% similarity and length equal 300 nts or greater. This non-redundant set of ESTs were annotated using BLASTX against a set of “best” proteins derived from six comprehensively annotated algal genomes^40,41,64–67^ and estimated levels of transcription in TPM (transcript per million) using Bowtie2^68^. Of the 59,571 ESTs, 20,096 (33.7% of non-redundant ESTs) were found to possess at least one best-hit from the database of “best” proteins, 12,609 (62.7% of annotated ESTs) of which showed no missing data from three repeated samples under both ND and NR conditions. A set of 12,609 annotated high-quality ESTs were used in this analysis (see Supplemental Table S1). Differentially transcribed ESTs were estimated using RSEM^69^ and EdgeR^70^ based on a cutoff of level change > 4-fold and FDR-corrected p-value < 0.01. The raw datasets were submitted to NCBI GEO database with an accession number of GSE118760 (https://www.ncbi.nlm.nih.gov/geo/query/acc.cgi?acc = GSE118760).

### Pathway-based sliding window analysis

Assuming a subset of ESTs associated with a particular metabolic pathway that is regulated by a common network would exhibit a similar transcription level (or ratio), the pathway-based sliding window methodology is to identify those potentially transcription regulatory networks^21,22^. First, all ESTs were sorted based on rank by level or ratio. A window with the size of 10% of all EST was moved along the sorted ESTs from high level to low level with a step of 5% of all EST. Thus, 19 windows covering the entire set of ESTs were set. Occurrence of ESTs associate with various KEGG pathways were counted and compared to the background level (number of ESTs associated with a particular pathway was divided by the total number of ESTs). A threshold (occurrence frequency > 2-fold compared to background, p-value < 0.05 after Bonferroni modification^71,72^ was set for potential functional regulation.

### Analysis of transcription levels of ESTs encoding enzymes associated with metabolic pathways

EC number of enzymes for unambiguity was used in analysis of direction of metabolic flux. Many enzymes were found to have be encoded by multi-copy genes that were summed in analysis. However, gene copy number and alternatively spliced transcription isoforms were indicated together with the number of ESTs that exhibited differential transcription (level change > 1.5-fold, p-value < 0.05) between ND and NR conditions.

### Lipid extraction and fatty acid methyl ester (FAME) preparation

Cell lipids were extracted using chloroform-methanol solution (2:1 v/v). The organic phase was transferred to a fresh tube blowing with nitrogen gas to evaporate organic solvent. The resulting lipid was weighted as quantity of total lipids and resuspended in hexane to a desired concentration. For analysis of fatty acid composition, one part of the extracted lipids was transesterified with methanol to generate fatty acid methyl ester (FAME) according to a published protocol^73^. FAME was weighted as quantity of acyl-lipids.

### Gas chromatography (GC) and GC coupled with mass spectrometry (GC-MS) analyses

GC analysis was applied to quantify various fatty acid molecular species as FAMEs once they were determined using GC-MS analysis. To determine the FAME species, 1 μl FAMEs was directly injected into the injection port of gas chromatograph (Shimadzu 2010Plus GC system, Shimadzu Co., Tokyo, Japan) coupled with a mass spectrometer system (MS) (Shimadzu QP2020 with quadrupole analyzer). The GC was operated on an Rtx-5MS GC column (30 m × 0.25 mm, id. with 0.25 μm film thickness of 5%-phenyl-methylpolysiloxane) (Restek Co., Bellefonte, PA, USA) and helium (purity 99.999%) was used as the carrier gas. The temperature of the injection port was set to 260 °C while the sample injection was made in splitless mode with a purge flow 50 mL min^−1^ for 1 min. The temperature program was started with an initial temperature at 160 °C, then 2 °C min^−1^ to 230 °C for 10 min. The mass spectrometer was operated in electron ionization (EI) mode with the ion source temperature at 230 °C. The electron energy was 70 eV. Full-scan MS data were acquired in the range of 50–500 m/z to obtain the fragmentation spectra of FAMEs. The LabSolutions (Shimadzu Co.) was used to determine all the peaks in raw GC chromatogram. Library search was done for all the peaks using the National Institute of Standards and Technology NIST/ EPA/NIH (NIST 14 Library).

To quantify various FAME species, 1 μL of FAMEs was directly injected into the injection port of gas chromatograph (Shimadzu Co., Japan) equipped with flame ionization detector (FID) and Rtx-5 column (30 m × 0.32 mm, id. with 0.25 mm film thickness) (Restek Co., Bellefonte, PA, USA). The sample injection was made in split mode and the split ratio was 20:1. The temperature of the injection port and detector temperature were set to 260 and 230 °C, respectively. The temperature program was started with an initial temperature at 160 °C, then 2 °C min^−1^ to 230 °C for 10 min. Nitrogen was used as carrier gas, and its flow rate was 30 mL min^−1^. Hydrogen gas flow rate and air flow rate were 40 and 400 mL min^−1^, respectively.

### Liquid chromatography coupled with tandem mass spectrometry (LC-MS/MS) analysis

To determine lipid classes and the associated fatty acid species, 10 mg freeze-dried PJ12 cell sample were used to extract lipids as above mentioned. Lipidomic analysis was performed with a mass spectrometer equipped with a RS 3000 UPLC system (Thermo Orbitrap Elite, Thermo Fisher, Waltham, MA, USA). The spray capillary voltage was 3.2 KV for the negative ion mode and 3.0 KV for the positive ion mode. Lipid extracts were separated at 45 °C on a Kinetex C18 column (1.9 mm, 2.10 × 100 mm, phenomenex) for positive- and negative-mode mass spectrometry analysis. The mobile phases were acetonitrile: water (60:40) (A) and acetonitrile: isopropanol (10:90) (B) containing 0.1% formic acid and 10 mM ammonium acetate; the LC gradients were as follows: 0 min, 70% A and 30% B; 2 min, 70% A and 30% B; 20 min, 0 A and 100% B; 40 min, 0% A and 100% B; 40.01 min, 70% A and 30%B; and 45 min, 70% A and 30% B. The flow rate was 0.4mL min^−1^. Relative quantification was achieved with LipidSearch (Thermo Fisher Scientific Inc) on the basis of intensity values of extracting masses of different lipids previously identified.

PC, PG and PE were analyzed as [M + H]^+^ ion and TAG and DAG as [M + NH_4_]^+^ at the positive mode, while DGDG, MGMG, FA, LPC, LPE and LPG were detected in the form of [M-H]^−^ and MGDG and LPC as [M + HCOO]^−^ ion at the negative mode. Precursor ion and neutral loss scanning modes were employed to identify lipid species by the LipidSearch (Thermo Fisher Scientific Inc.). For most lipid classes, fatty acid at the *sn*1 or *sn*2 position of the glycerol backbone was determined by following the published methods^8,35^. For some undetermined species, fatty acids at the *sn* positions were estimated by comparing the relative intensities of the MS/MS fatty acid fragments to those of the characterized species of the same class under the same LC-MS/MS conditions. A total of 80 lipid species within eleven lipid classes is available in Supplemental Table 2.

## Supplementary information

Supplementary information

Sup_Table

## Data Availability

The datasets generated during and/or analysed during the current study are available from the corresponding author on reasonable request.
