# Supplementary material for: Transcriptomic and lipidomic analysis of an EPA-containing Nannochloropsis sp. PJ12 in response to nitrogen deprivation
Source: Sci Rep. 2019 Mar 14;9:4540. doi: 10.1038/s41598-019-41169-2 (PMC6418175; doi:10.1038/s41598-019-41169-2)
Supplement: Supplementary file 1 — Supplementary information [file 41598_2019_41169_MOESM1_ESM.pdf]

Transcriptomic and lipidomic analysis of an EPA-containing *Nannochloropsis* sp. PJ12 in response to nitrogen deprivation

Jibei Liang[1], Fang Wen[1], and Jianhua Liu[1,2*]

[1]Ocean College, Zhejiang University, Zhoushan, ZJ316000, China

[2]Ocean Research Center of Zhoushan, Zhoushan, ZJ316021, China

[*]Corresponding author

Send all correspondences to:

Jianhua Liu, Professor

Ocean College, Zhejiang University

Zhoushan Campus, Marine Science Building, Room 379

1 Zheda Road, Dinghai District

Zhoushan, Zhejiang Province 316000, China

Tel: 86-580-2186223

Fax: 86-580-2186317

Email: liujh2013@zju.edu.cn

ORCID ID: 0000-0003-1165-9348

Supplemental Figure S1. Characteristics of PJ2 transcriptome. (A) EST length (in bps) distribution. X- and Y-axes indicate binned length and numbers of ESTs, respectively. (B) EST transcription level (in TPM) distribution. X- and Y-axes indicate binned level and numbers of ESTs. (C) Top 10 GO Biological process (BP) functions in the transcriptome. Numbers of GO function associated ESTs are shown. (D) Top 10 KEGG Metabolic pathways (MP) in the transcriptome. Numbers of MP associated ESTs are shown.

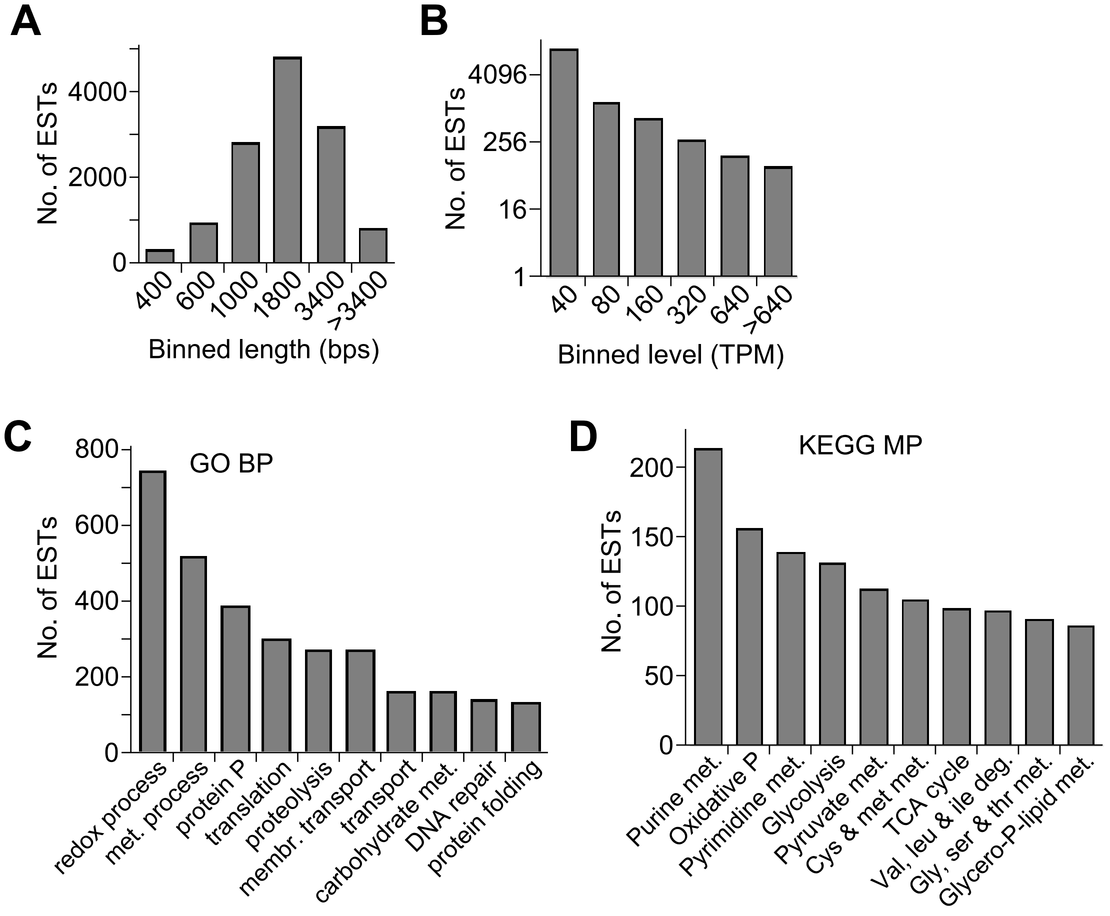

Supplemental Figure S2. Change of FAME profiles under ND and NR conditions. GC spectra of ND and NR are indicated. Number of lipid species are shown in Figure 2D.

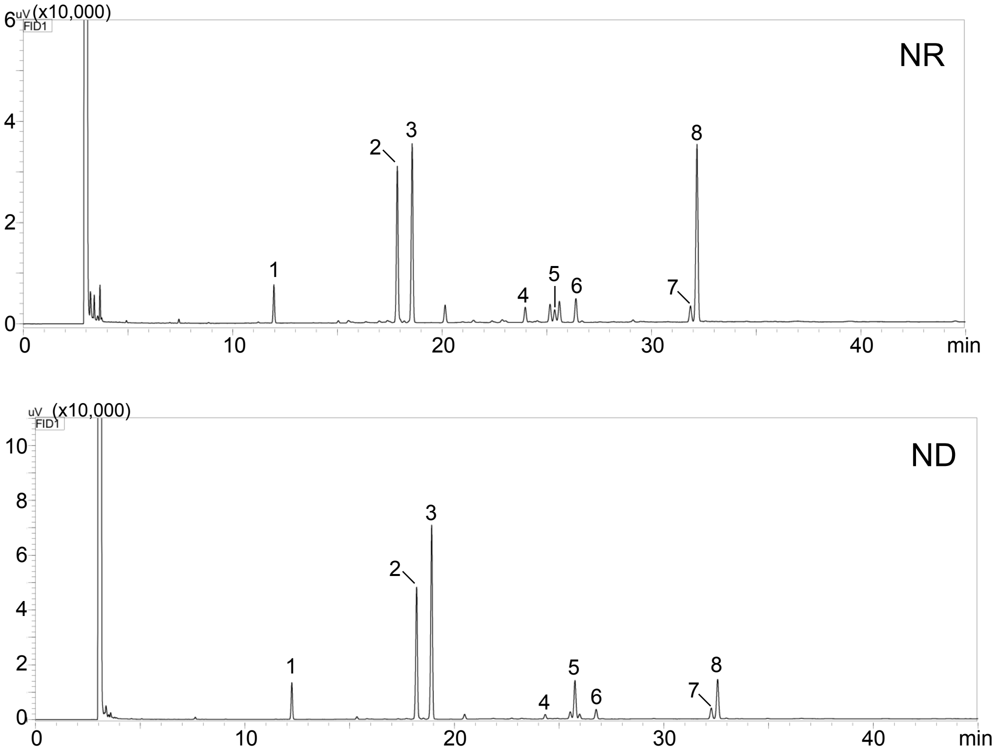

Supplemental Method S1. Analysis of homologous gene sequences between transcriptome of PJ12 and genomes of four *Nannochloropsis* spp.

To analyze PJ12 homologues in other *Nannochloropsis* spp., 20,096 non-redundant ESTs were subjected to BLASTX analysis (http://blast.ncbi.nlm.nih.gov) against protein sequences or CDSs pooled from four *Nannochloropsis* spp.: *N. oceanica* CCMP1779[1], *N. oceanica* LAMB0001 [2], *N. gaditana* CCMP526 [3], and *N. gaditana* B-31 [4]. All CDSs sequences met the cutoff of e-value < 1e-07 were collected. Unique numbers of CDS sequences from each strain were reported as number of homologues in various *Nannochloropsis* spp. found for PJ12. The result is shown as follows:

**Isolate**	**Total CDS #**	**Hom.[a] CDS #**	**Percent**
CCMP1779	12,012	11,341	92.3%
LAMB0001	11,129	10,271	92.3%
CCMP526	9,053	7,433	82.1%
B-31	21,957	8,863	40.4%

Note: a, Hom. stands for homologous.

References

1 Vieler, A. *et al.* Genome, functional gene annotation, and nuclear transformation of the heterokont oleaginous alga Nannochloropsis oceanica CCMP1779. *PLoS Genet* **8**, e1003064, doi:10.1371/journal.pgen.1003064 (2012).

2 Pan, K. *et al.* Nuclear Monoploidy and Asexual Propagation of Nannochloropsis Oceanica (Eustigmatophyceae) as Revealed by Its Genome Sequence(1). *J Phycol* **47**, 1425-1432, doi:10.1111/j.1529-8817.2011.01057.x (2011).

3 Radakovits, R., Jinkerson, R. E., Darzins, A. & Posewitz, M. C. Genetic engineering of algae for enhanced biofuel production. *Eukaryot Cell* **9**, 486-501, doi:10.1128/EC.00364-09 (2010).

4 Corteggiani Carpinelli, E. *et al.* Chromosome Scale Genome Assembly and Transcriptome Profiling of Nannochloropsis gaditana in Nitrogen Depletion. *Molecular Plant* **7**, 323-335, doi:https://doi.org/10.1093/mp/sst120 (2014).
